# A novel interplay between bacteria and metabolites in different early-stage lung cancer: an integrated microbiome and metabolome analysis

**DOI:** 10.3389/fonc.2024.1492571

**Published:** 2025-01-07

**Authors:** Xiaoqian Zhai, Dongqi Lin, Yi Shen, Ni Zhai, Fan Yu, Jiabi Zhang, Yiyun Lin, Yuqing Wang, Qinghua Zhou, Xi Zheng

**Affiliations:** ^1^ Department of Medical Oncology, Cancer Center, West China Hospital, Sichuan University, Chengdu, Sichuan, China; ^2^ Lung Cancer Center, West China Hospital, Sichuan University, Chengdu, Sichuan, China; ^3^ Department of Thoracic Surgery, West China Hospital, Sichuan University, Chengdu, Sichuan, China; ^4^ Department of Thoracic Surgery, West China School of Public Health and West China Fourth Hospital, Sichuan University, Chengdu, Sichuan, China; ^5^ Neurosurgery Intensive Care Unit, The 987th Hospital of the Joint Logistics Support Force of the Chinese People's Liberation Army, Baoji, Shanxi, China; ^6^ Department of Nutrition and Integrative Physiology, College of Health, University of Utah, Salt Lake City, UT, United States; ^7^ Graduate School of Biomedical Sciences, MD Anderson Cancer Center UT Health, Houston, TX, United States; ^8^ Graduate School of Biomedical Sciences, Baylor College of Medicine, Houston, TX, United States

**Keywords:** early-stage lung cancer, microbiome, metabolome, correlation analysis, carcinogenesis

## Abstract

**Background:**

The carcinogenesis mechanism of early-stage lung cancer (ESLC) remains unclear. Microbial dysbiosis is closely related to tumor development. This study aimed to analyze the relationship between microbiota dysbiosis in ESLC.

**Methods:**

We investigated a total of 108 surgical specimens of lung nodules, including ground glass nodules (GGN) diagnosed as lung adenocarcinoma (*n* = 25), solid nodules (SN) diagnosed as lung adenocarcinoma (*n* = 27), lung squamous carcinoma (LUSC) presenting as solid nodules (*n* = 26), and benign pulmonary nodules (BPD) (*n* = 30) that were collected. 16S rDNA amplicon sequencing and non-targeted metabolomics analysis were performed in all of the specimens.

**Results:**

We found a significantly lower microbiota richness in SN than in the GGN and LUSC. *Ralstonia* may be an important flora promoting the development of early lung adenocarcinoma, while *Feacalibacterium* and *Blautia* play a protective role in the progression of GGN to SN. *Akkermansia*, *Escherichia-shigella*, and *Klebsiella* exhibited high abundance in early lung squamous carcinoma. Compared with BPD, the differential metabolites of both early adenocarcinomas (SN and GGN) are mainly involved in energy metabolic pathways, while early LUSC is mainly involved in glutathione metabolism, producing and maintaining high levels of intracellular redox homeostasis. A correlation analysis revealed that different microbiota in GGN may function in energy metabolism via N-acetyl-1-aspartylglutamic acid (NAAG) when compared to BPD, while creatine and N-acetylmethionine were the main relevant molecules for the function of differential microbiota in LUSC.

**Conclusion:**

Our study identified that early-stage lung adenocarcinoma and squamous carcinoma differ in microbial composition and metabolic status. *Ralstonia* may be an important flora promoting the development of early lung adenocarcinoma, while *Feacalibacterium* and *Blautia* play a protective role in the progression of GGN to SN. Conversely, *Akkermansia*, *Escherichia-shigella*, and *Klebsiella* exhibited high abundance in early lung squamous carcinoma. The metabolites of both early adenocarcinomas (SN and GGN) are mainly involved in energy metabolic pathways, while early LUSC is mainly involved in glutathione metabolism. Our study provides new insights into the carcinogenesis of ESLC.

## Introduction

Microbes are an important human body component, accounting for 1%–3% of the body mass (1). A growing body of research suggests that microbiota exerts an important role in tumor development (2, 3). Microbes and their derivatives can modulate and disrupt the body’s genes under specific conditions, leading to the occurrence and development of disease (4). The lung microbiota consists of bacteria, fungi, and viruses that live in wonderful balance with the host (5, 6). In the case of microbial dysbiosis in the lung, a high abundance of pathogenic bacteria may increase the host’s susceptibility to carcinogenic events (5, 7, 8). In addition, bacterial metabolites and toxins of pathogenic microbes can influence the activation of molecular pathways associated with oncogenic signaling and thus promote tumor progression (9, 10)—for example, *Prevotella*, *Streptococcus*, and *Veillonella* can induce PI3K and ERK signaling pathways in airway epithelial cells (11). Another recent study found that local microbiota dysbiosis in the lung can activate lung-resident γδ T cells, produce IL-17 and other effector molecules, and influence the immune status of the lung to promote lung adenocarcinoma progression (12). So, microbial imbalance in the lung may be associated with tumorigenesis and progression through multiple pathways.

With the widespread use of high-resolution computed tomography (CT) in lung cancer screening, pulmonary nodules’ diagnosis rate has significantly increased (13, 14). Benign nodules account for 95% of pulmonary nodules and are most commonly granulomas or intrapulmonary lymph nodes (15). In contrast, 85% of malignant pulmonary nodules are ground glass nodules (GGNs), which are less aggressive than solid nodules (SN) (16, 17). Although these patients can undergo surgical resection, a high risk of relapse still exists, leading to a greater impact on the survival and prognosis of patients (18). However, the oncogenic mechanism of these early-stage lung cancers (ESLC) and the mechanism leading to the progression of GGNs to SN remain unclear.

Few studies have directly examined the association of microbial dysbiosis in the lung with carcinogenesis of ESLC. So far, only one study directly investigated lung microbiota dysbiosis with GGN and SN occurrence by 16s rRNA sequencing using tumor tissue specimens (19). However, the sample size of the pulmonary nodules in that study was small; no further analysis of the oncogenic effects of microbial-derived metabolites was performed. Therefore, herein we used a large sample of lung nodule tissues to investigate the molecular mechanisms of lung microbiota dysbiosis in the development of ESLC with different characteristics by microbiome, metabolome, and correlative analysis.

## Methods

### Participants

A total of 108 patients were included in this study. The inclusion criteria were as follows: age >18 years, diagnosed with pulmonary nodules using chest CT, and no treatment received prior to surgery. The exclusion criteria were as follows: stage IV lung cancer; use of antibiotics, probiotics, prebiotics, or synbiotics in the previous 6 months; and chemotherapy, radiotherapy, or other biological therapy prior to radical resection of lung cancer. The clinical and pathological staging, respectively, was performed by three pathologists affiliated with West China Hospital of Sichuan University, in accordance with the 8th edition of the Union for International Cancer Control (UICC) TNM staging system for lung cancer (20).

### 16S rDNA amplicon sequencing

The lung cancer tissue was isolated during surgery, and liquid nitrogen was used for rapid preservation of lung cancer specimens. Obtaining surgical specimens, genomic DNA was extracted. Different regions of the 16S rDNA were amplified and purified. Amplicon: 16S rDNA genes in distinct regions (16S V4/16S V3/16S V3-V4/16S V4-V5) were amplified with a specific primer (e.g., 16S V4: 515F- 806R) and barcodes. All PCR mixtures contained 15 µL of Phusion^®^ High-Fidelity PCR Master Mix (New England Biolabs), 0.2 µM of each primer, and 10 ng target DNA, and the cycling conditions consisted of a first denaturation step at 98°C for 1 min, followed by 30 cycles at 98°C (10 s), 50°C (30 s), and 72°C (30 s), and a final 5-min extension at 72°C. Purification: Mix an equal volume of 1X loading buffer (contained SYB green) with PCR products and perform electrophoresis on 2% agarose gel for DNA detection. The PCR products were mixed in equal proportions, and then Qiagen Gel Extraction Kit (Qiagen, Germany) was used to purify the mixed PCR products. Different regions of the 16S rDNA were amplified and purified. Observed_species (the number of observed species) and the Shannon index were calculated in QIIME. Principal coordinates analysis (PCoA) was carried out to the differences in community structure between different groups. *T*-test method using the R software (version 3.5.3) was used to test the significance of the differences in the species composition and community structure of the grouped samples (abundance >0.001, *P*-value <0.05, adjusted *P*-value <0.05).

### Tissue sample

Tissues (100 mg) were individually ground with liquid nitrogen, and the homogenate was resuspended with prechilled 80% methanol by well vortex. The samples were incubated on ice for 5 min and then were centrifuged at 15,000 *g* at 4°C for 20 min. Some of the supernatant was diluted to a final concentration containing 53% methanol by LC-MS grade water. The samples were subsequently transferred to a fresh Eppendorf tube and then were centrifuged at 15,000 *g* at 4°C for 20 min. Finally, the supernatant was injected into the LC–MS/MS system analysis.

### UHPLC–MS/MS analysis

UHPLC–MS/MS analyses were performed using a Vanquish UHPLC system (ThermoFisher, Germany) coupled with an Orbitrap Q ExactiveTMHF-X mass spectrometer (Thermo Fisher, Germany) by Novogene Co., Ltd. (Beijing, China). The samples were injected onto a Hypesil Gold column (100 × 2.1 mm, 1.9 μm) using a 17-min linear gradient at a flow rate of 0.2 mL/min. The eluents for the positive polarity mode were eluent A (0.1% FA in water) and eluent B (methanol). The eluents for the negative polarity mode were eluent A (5 mM ammonium acetate, pH 9.0) and eluent B (methanol). The solvent gradient was set as follows: 2% B, 1.5 min; 2%–100% B, 3 min; 100% B, 10 min; 2%–100% B, 10.1 min; 2% B, 12 min. Q ExactiveTM HF-X mass spectrometer was operated in positive/negative polarity mode with a spray voltage of 3.5 kV, capillary temperature of 320°C, sheath gas flow rate of 35 psi and aux gas flow rate of 10 L/min, S-lens RF level of 60, and aux gas heater temperature of 350°C.

### Data processing and metabolite identification

The raw data files generated by UHPLC–MS/MS were processed using Compound Discoverer 3.1 (CD3.1, ThermoFisher) to perform peak alignment, peak picking, and quantitation for each metabolite. The main parameters were set as follows: retention time tolerance, 0.2 min; actual mass tolerance, 5 ppm; signal intensity tolerance, 30%; signal/noise ratio, 3; and minimum intensity, etc. After that, the peak intensities were normalized to the total spectral intensity. The normalized data was used to predict the molecular formula based on additive ions, molecular ion peaks, and fragment ions. Then, the peaks were matched with mzCloud (https://www.mzcloud.org/), mzVault, and MassList database to obtain accurate qualitative and relative quantitative results. Statistical analyses were performed using the statistical software R (R version R-3.4.3), Python (Python 2.7.6 version), and CentOS (CentOS release 6.6). When data were not normally distributed, normal transformations were attempted using the area normalization method.

### Data analysis

These metabolites were annotated using the KEGG database (https://www.genome.jp/kegg/pathway.html), HMDB database (https://hmdb.ca/metabolites), and LIPIDMaps database (http://www.lipidmaps.org/). Principal component analysis (PCA) and partial least squares—discriminant analysis (PLS-DA) were performed at metaX (a flexible and comprehensive software for processing metabolomics data). We applied univariate analysis (*t*-test) to calculate the statistical significance (*P*-value). The metabolites with VIP >1 and *P*-value <0.05 and fold change ≥2 or FC ≤0.5 were considered to be differential metabolites. Volcano plots were used to filter metabolites of interest which based on log2(foldchange) and -log10(p-value) of metabolites by ggplot2 in R language.

For clustering heat maps, the data were normalized using z-scores of the intensity areas of differential metabolites and were plotted by using Pheatmap package in R language. The correlation between differential metabolites was analyzed by cor () in R language (method=pearson). Statistically significant correlations between differential metabolites were calculated by cor.mtest() in R language. *P*-value <0.05 was considered as statistically significant, and correlation plots were plotted by corrplot package in R language. The functions of these metabolites and metabolic pathways were studied using the KEGG database. The metabolic pathways’ enrichment of differential metabolites was performed; when the ratio was satisfied by *x*/*n* > *y*/*N*, metabolic pathway was considered as enrichment, and when the *P*-value of metabolic pathway <0.05, metabolic pathways were considered to have statistically significant enrichment.

### Combined microbiome–metabolome analysis

At the genus level, the different microbiota from 16S rDNA analysis and the different metabolites from metabolomics analysis were correlated based on Pearson correlation coefficients.

### Random forest prediction model

A random forest model was built based on differential microbiota and differential metabolites in different ESLC and trained by performing five-fold cross-validation using the R package. Model performance was evaluated using the area under the ROC curve (AUC).

## Results

### Baseline clinical data and prognosis of pulmonary nodules with different pathological types

**Figure 1:**
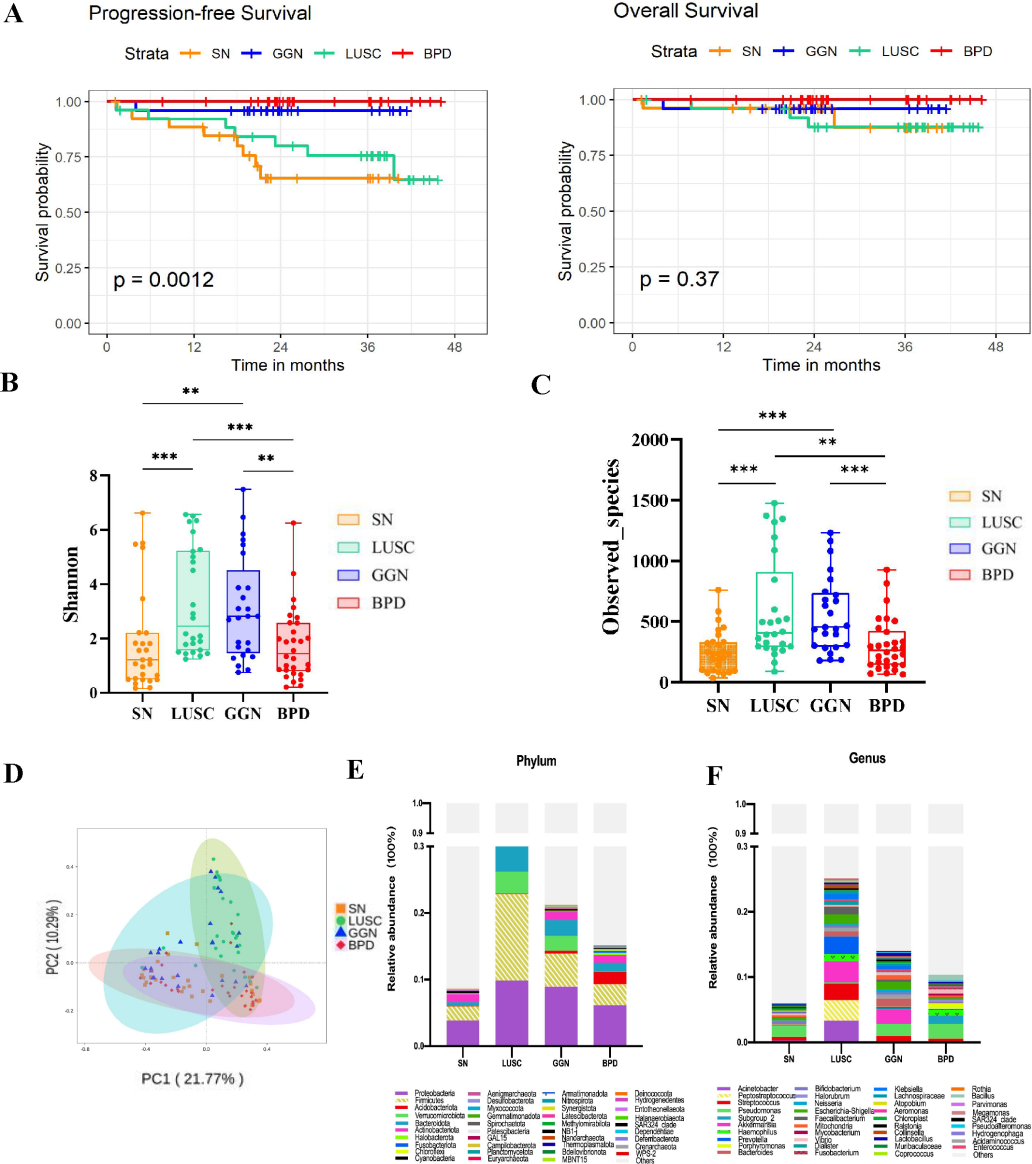
Comparison of the microbiome characteristics of GGN, SN, and LUSC. **(A)** Kaplan–Meier survival curves comparing the PFS and OS of GGN, SN, and LUSC. **(B, C)** Shannon and Observed_species indices comparing the microbial diversity of GGN, SN, and LUSC. **(D)** Principal coordinates analysis (PCoA) to compare the inter- and intra-group heterogeneity of GGN, SN, and LUSC and compare the relative microbial abundance of GGN, SN, and LUSC at the phylum level **(E)** and at the genus level **(F)**. GGN, patients with ground glass nodules with a pathological diagnosis of adenocarcinoma; SN, patients with solid nodules with a pathological diagnosis of adenocarcinoma, LUSC, patients with a pathological diagnosis of squamous lung cancer but with radiologically solid nodules; BPD, group of patients with benign pulmonary nodules (*P < 0.05; **P < 0.01; ***P < 0.001).

A total of 108 patients were included in this study. The participants were divided into four major groups according to the post-surgical pathological diagnosis: GGN patients diagnosed with lung adenocarcinoma (*n* = 25), SN patients diagnosed as lung adenocarcinoma (*n* = 27), lung squamous carcinoma (LUSC) group with a solid nodule on imaging (*n* = 26), and benign pulmonary nodule (BPD) group (*n* = 30). The mean age at diagnosis in the BPD group (54.27 ± 10.39) was lower compared to the three groups (GGN group, 64.36 ± 9.82, SN group, 59.40 ± 9.48; LUSC group, 60.00 ± 7.78), according to the clinical baseline data collected and counted from the enrolled patients (**Table 1**). There were 58 males and 50 females; the LUSC group included only male patients. There was no difference in the male-to-female ratio in the other groups. The progression-free survival (PFS) and overall survival (OS) in the four groups with different pathological types of pulmonary nodules (PFS and OS calculated from diagnosis to the last follow-up visit on 2022.12.31) revealed that the SN group with a diagnosis of lung adenocarcinoma had the worst prognosis (**Figure 1A**).

**Table 1:** Clinicopathological characteristics of the patients.

Characteristics	SN	GGN	LUSC	BPD
Total number	27	25	26	30
Gender, number				
Female	16	15	0	19
Male	11	10	26	11
Age (mean ± SD)	59.40 ± 9.48	64.36 ± 9.82	60.00 ± 7.78	54.27 ± 10.39
Smoking history, number				
Present/ex-smoker	8	8	25	8
Non-smoker	19	17	1	22
Lesion location, number				
Right upper lobe	8	12	8	12
Right middle lobe	3	1	2	3
Right lower lobe	6	4	9	6
Left upper lobe	5	5	7	3
Left lower lobe	5	3	0	6
pT stage, number				
T1a	0	0	0	–
T1b	4	13	0	–
T1c	9	8	2	–
T2a	10	3	10	–
T2b	2	1	1	–
T3	2	0	8	–
T4	0	0	5	–
pN stage, number				
N0	20	25	16	–
N1	3	0	4	–
N2	4	0	6	–
pTNM stage, number				
IA1	0	0	0	–
IA2	4	13	0	–
IA3	7	8	0	–
IB	9	3	7	–
IIA	0	1	1	–
IIB	3	0	8	–
IIIA	1	0	6	–
IIIB	3	0	4	–
Tumor differentiation, number				
Well differentiated	0	3	0	–
Moderately differentiated	8	15	12	–
Poorly differentiated	16	1	12	–
NA	3	6	2	–

SN, solid nodule; GGN, ground glass nodule; LUSC, lung squamous cell carcinoma; BPD, benign pulmonary disease.

### The microbial diversity and composition of GGN, SN, and LUSC are different

Shannon index was positively correlated with richness and evenness of the microbiota of pulmonary nodules in each group. The Shannon index of the GGN group and LUSC group were higher than those of the SN group (*P*=0.0011, *P*=0.0002), while there was no significant difference in the Shannon index between the SN group and BPD group (*P*=0.6275) (**Figure 1B**). Next, Observed_species was used to detect the number of species in different pulmonary nodule group. The number of species detected in the GGN group and LUSC group was significantly higher than that in the SN group (*P*= 0.0001, *P*= 0.0001) (**Figure 1C**). Significant clustering was detected for the Principal Co-ordinates Analysis (PCoA) analysis among BPD, GGN, SN and LUSC group. PCoA analysis aims to analyze the characteristics of different ESLC microbiota composition (**Figure 1D**). At the phylum level, we found that *Proteobacteria*, *Firmicutes* and *Bacteroidota* were the dominant flora in four groups of pulmonary nodules (**Figure 1E**). However, at the genus level, we found that *Pseudomonas* was dominant flora in GGN, SN, and BPD, while *Acinetobacter*, *Akkermansia*, and *Peptostreptococcus* in LUSC (**Figure 1F**).

### Analysis of the differential microbiota of GGN, SN, and LUSC

**Figure 2:**
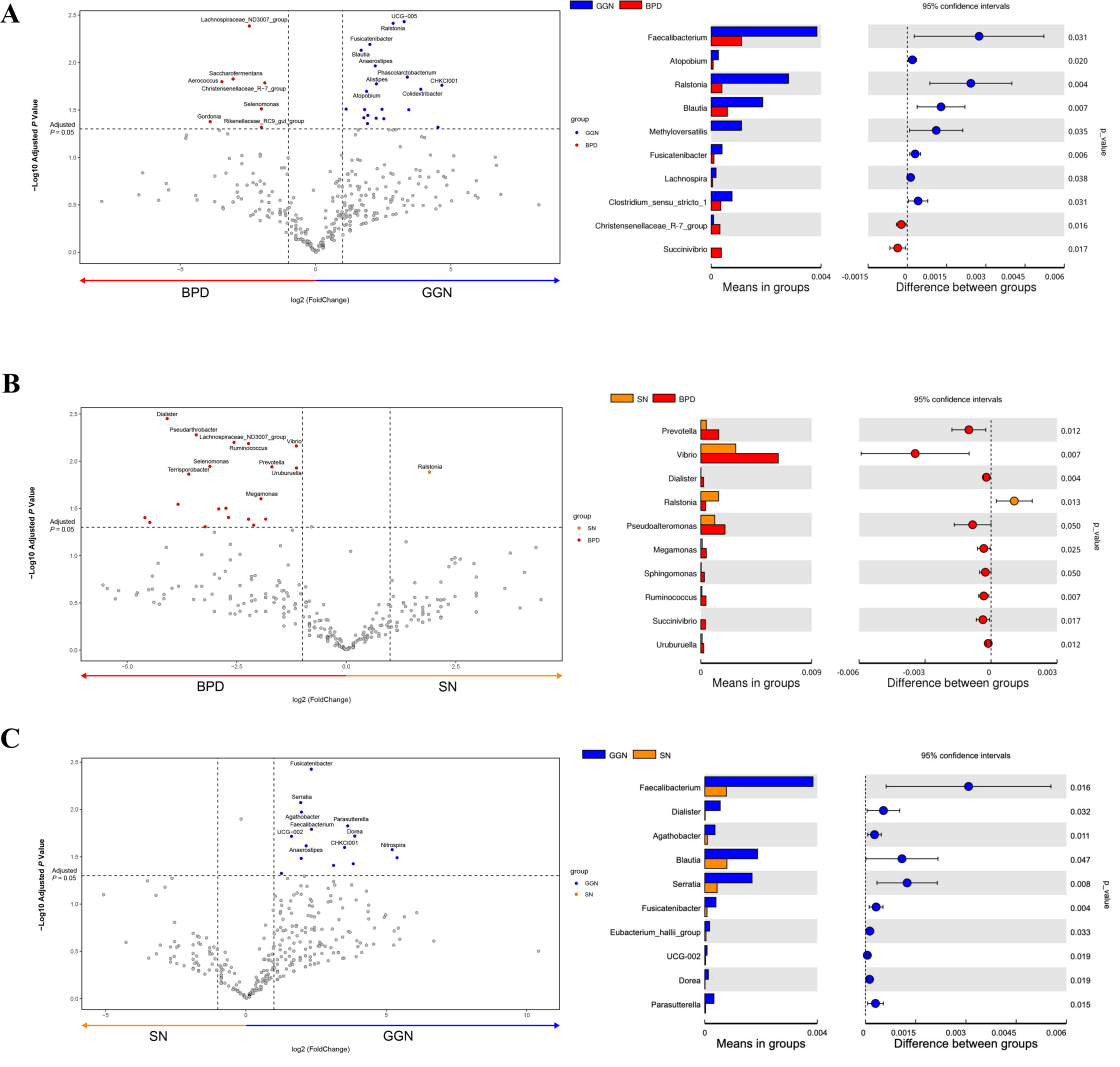
Comparison of differential microflora between GGN, SN, and BPD, respectively. Volcano plot using *T*-test analysis at the genus level, showing the differential flora (fold change > 2). Bar graph showing the mean abundance and *P*-value of GGN vs. BPD **(A)**, SN vs. BPD **(B)**, and GGN vs. SN **(C)**.

At the genus level, we found *Ralstonia*, *Blautia*, and *Faecalibacterium* significantly increased in the GGN group compared to BPD group (**Figure 2A**). *Ralstonia* also exhibited higher abundance in SN group than in BPD group (**Figure 2B**). Further, we compared GGN and SN (two groups early adenocarcinoma) and found that *Feacalibacterium*, *Serratia*, and *Blautia* were enriched in the GGN and decreased in the SN group (**Figure 2C**). When compared LUSC with BPD (**Supplementary Figure S1A**) or lung adenocarcinoma (SN) (**Supplementary Figure S1B**), respectively, *Akkermansia*, *Escherichia-shigella* and *Klebsiella* were found to be significantly enriched in the LUSC group at the genus level. The above findings suggest that the composition of the microbiota is highly variable in different pathological types of pulmonary nodules.

### Differential metabolites and differential metabolic pathways of GGN, SN, and LUSC

**Figure 3:**
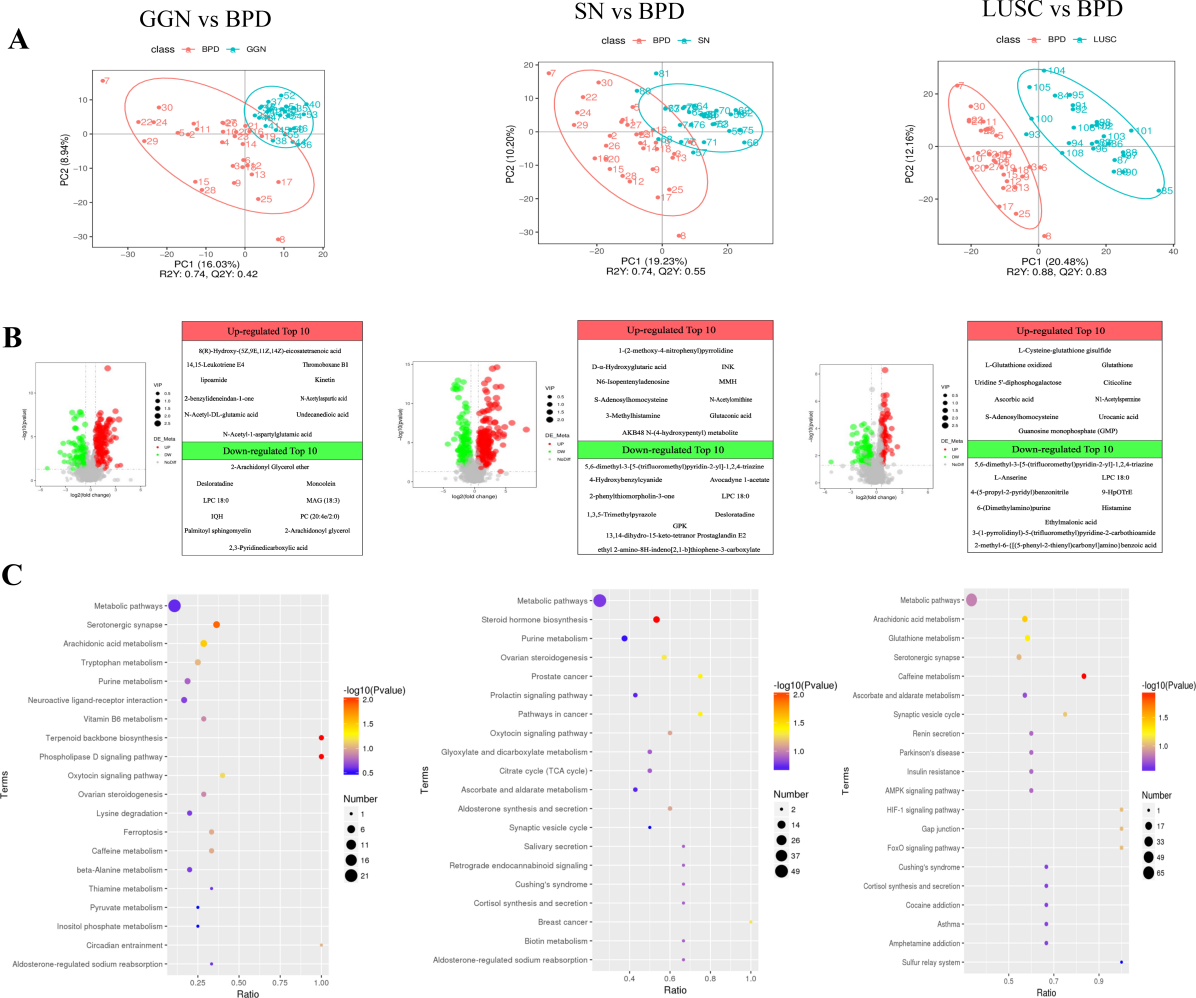
Differential metabolites and pathways of GGN, SN, and LUSC compared with BPD, respectively. **(A)** Partial least squares—discriminant analysis (PLS-DA) score scatterplot of GGN, SN, and LUSC when compared to BPD, respectively. R2Y greater than Q2Y indicates good model establishment. **(B)** Volcano plot showing the differential metabolites of GGN, SN, and LUSC when compared to BPD, respectively. Set thresholds: variable importance in the projection (VIP) >1.0, FC >1.5, or FC <0.667 and *P*-value <0.05. Red dots represent significant upregulation, and green dots represent significant downregulation. FC, fold change. **(C)** Bubble plots of KEGG enrichment of GGN, SN, and LUSC when compared to BPD, respectively. The more significant the *P*-value, the redder the color in the bubble. The larger the size of the dots, the more differential metabolites are present within this pathway.

**Figure 4:**
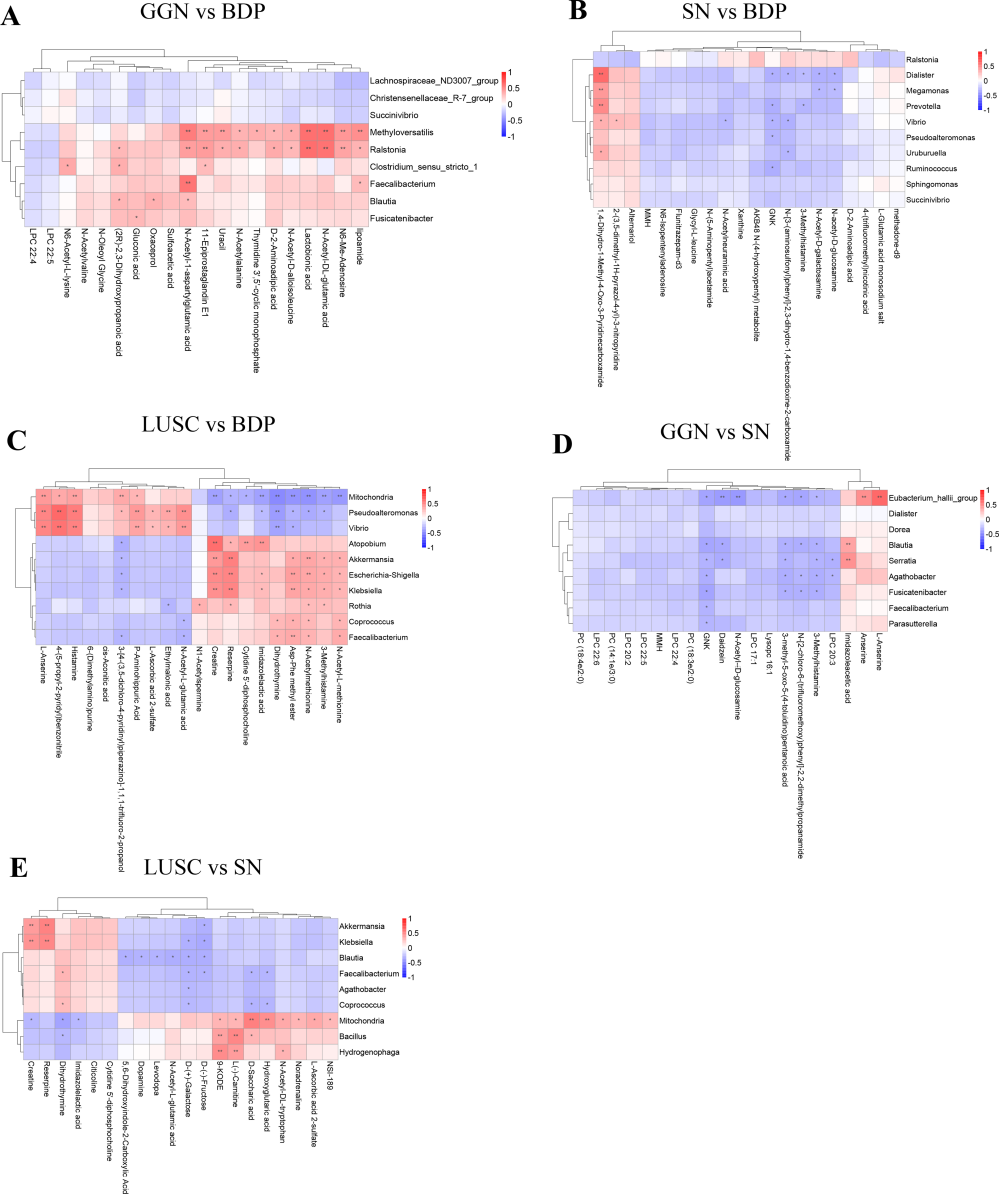
Association analysis between differential microbiota and metabolites of GGN, SN, and LUSC. Spearman’s correlation method was used to analyze the correlation between the top 20 differential microorganisms and 20 differential metabolites at the microbial genus level when GGN vs. BPD **(A)**, when SN vs. BPD **(B)**, when LUSC vs. BPD **(C)**, when GGN vs. SN **(D)**, and when SN vs. LUSC **(E)**. Horizontal coordinates represent differential metabolites. Vertical coordinates represent differential microorganisms. The red color represents a positive correlation between differential microorganisms and differential metabolites. The blue color represents a negative correlation. The lower the *P*-value, the more significant the correlation (*P < 0.05; **P < 0.01.

Microbiota often function through their derived metabolites; therefore, we further analyzed the differential metabolites in each group of pulmonary nodules. We validated the metabolomic data using the OPLS-DA model and found that the metabolic data were comparable between the lung nodule pairs (**Figure 3A**). When compared with BPD, the top 3 up-regulated metabolites in GGN were: 8(R)-Hydroxy-(5Z,9E,11Z,14Z)-eicosatetraenoic acid (fold change=4),14,15- Leukotriene E4 (fold change=4) and Thromoboxane B1 (fold change=4), while the top 3 up-regulated metabolites in the SN group were D-α-hydroxyglutaric acid (fold change=16), MMH (fold change=8), and glutaconic acid (fold change=8) (**Figure 3B**). Thus, KEGG enrichment analysis showed that the differential metabolites of GGN were mainly enriched in phospholipase D (PLD) signaling pathway and arachidonic acid metabolism, whereas the up-regulated metabolites of the SN group were mainly enriched in the steroid hormone biosynthesis pathway (**Figure 3C**). When compared LUSC with BPD, we found the top 3 up-regulated differential metabolites in LUSC were L-Glutathione oxidized (fold change=256), L-Cysteine-glutathione disulfide (fold change=64) and Glutathione (fold change=32) (**Figure 4C**), which were mainly enriched in the glutathione metabolism pathway (**Figure 3C**).

### Microbiome and metabolome association analysis results of GGN, SN, and LUSC

To measure the association between differential microbiota and metabolites in different pulmonary nodules, we found that differential microbes enriched in the GGN were positively correlated with N-Acetyl-1-aspartylglutamic acid (NAAG) and N-Acetyl-DL-glutamic acid compared to BPD (**Figure 4A**), while no metabolites was positively associated with differential microbiota in SN (**Figure 4B**). Furthermore, we compared the two groups of early adenocarcinoma (GGN and SN) and found that the differential microorganisms enriched in GGN were negatively correlated with GNK, 3-Methylhistamine (**Figure 4D**).

At final, when compared to BPD, our results showed differential microorganisms in LUSC were positively correlated with Creatine and N-Acetylmethionine (**Figure 4C**). However, when compared to SN, differential microorganisms in LUSC were positively correlated with Creatine but negatively correlated with D-(+)-Galactose (**Figure 4E**).

### Random forest model prediction of biomarkers in GGN, SN, and LUSC

**Figure 5:**
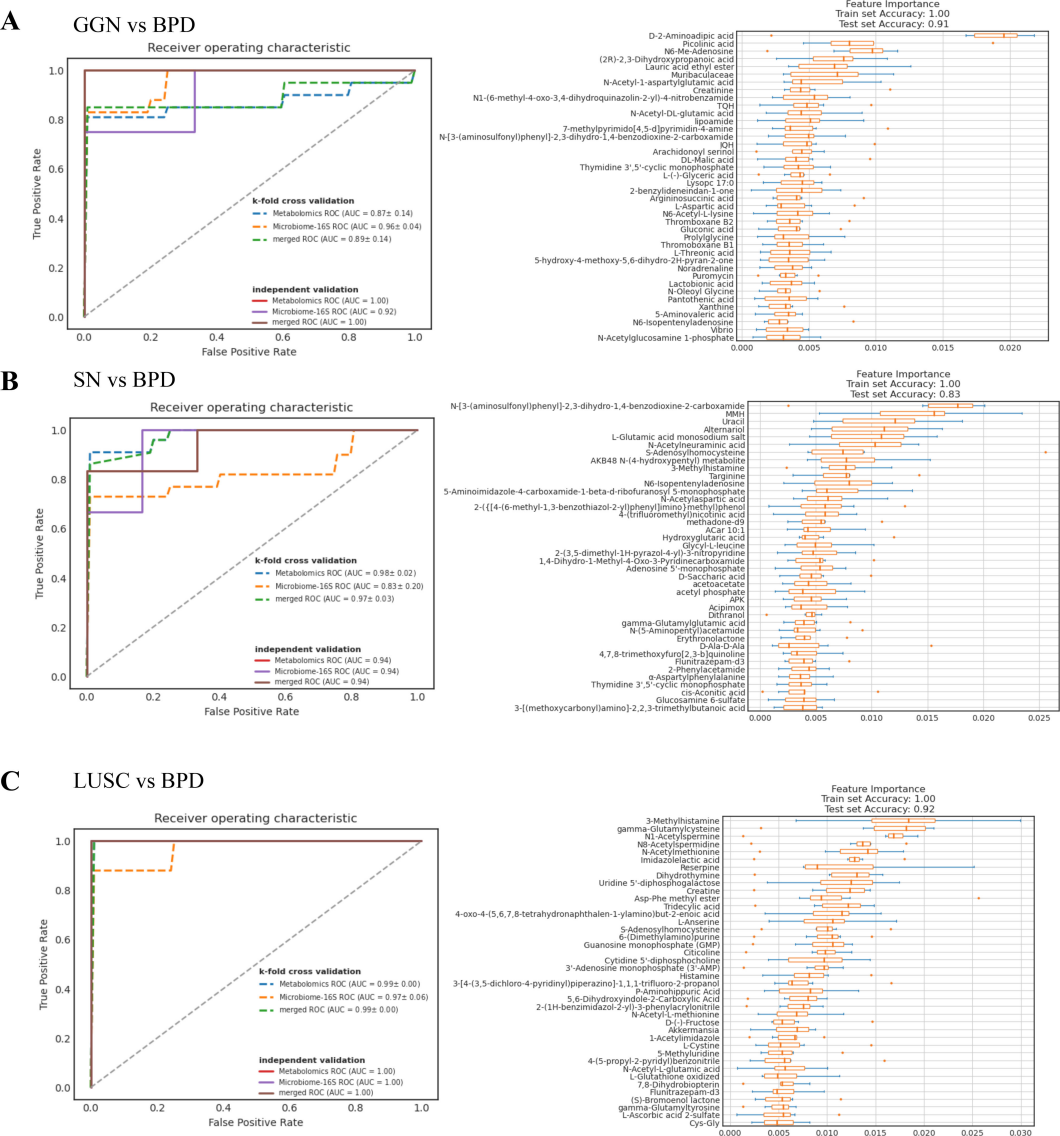
Random forest model predicts biomarkers for different pathological types of pulmonary nodules. **(A)** ROC curves of random forest models for GGN and BPD. The solid line represents the test set ROC, while the dashed line represents the k-repeat cross-validation training set ROC. The area under the curve is the AUC value. The closer the AUC value is to 1, the more accurate the model prediction is. Feature importance boxplot for GGN and BPD. The vertical coordinate is the feature importance, which is used to determine the contribution of metabolites or microorganisms in the model. The horizontal coordinate is the name of the metabolite or microorganism. **(B)** ROC plots of random forest models for SN and BPD. Feature importance boxplot for SN and BPD. **(C)** ROC plots of random forest models for LUSC and BPD. Feature importance boxplot for LUSC and BPD.

We constructed a five-fold cross-validated random forest classification model using GGN, SN and LUSC microbiota and metabolome features to find biomarkers for distinguishing malignant nodules from benign pulmonary nodules. The results of the model showed in the training set and the validation set the AUC based on the differential metabolite model or the differential microbial model, which was close to or equal to 1. Meanwhile, the ROC of the mixed differential microbial and differential metabolite model was close to or equal to 1. This suggested that the predictive ability of this model was reliable. Therefore, we found that D-2-aminoadipic acid, picolinic acid, and N6-Me-adenosine might distinguish GGN from BPD (**Figure 5A**), while MMH and uracil could distinguish SN from BPD (**Figure 5B**). Lastly, 3-methylhistamine and gamma-glutamylcysteine might be able to distinguish LUSC from BPD (**Figure 5C**).

## Discussion

To the best of our knowledge, this is the first study that explored ESLC development mechanisms by directly using tumor tissues to perform large-scale microbiome and metabolome sequencing. Our study found that *Ralstonia* may be an important flora promoting the development of early lung adenocarcinoma, while *Feacalibacterium* and *Blautia* play a protective role in the progression of GGN to SN. The metabolites of both early adenocarcinomas (SN and GGN) are mainly involved in energy metabolic pathways, while early LUSC are mainly involved in glutathione metabolism, producing and maintaining high levels of intracellular redox homeostasis. Our study provides new insights into the carcinogenesis of ESLC.

Decreased microbiome diversity and richness in lung cancer tissues are associated with poor prognosis and poor survival of patients (21, 22). Our study found that SN had the worst prognosis as well as the lowest microbiome richness when compared with other groups. This is consistent with previous findings that biodiversity is significantly lower in highly invasive SN adenocarcinomas than in inert tumor GGN (19). However, we also found no significant difference in biodiversity between the poorly prognostic SN group and BPD group, which is not consistent with some previous studies. Wang et al. analyzed the tissues of lung cancer and healthy individuals and found that lung cancer patients had decreased microbiota diversity compared to those with normal tissue (23) Zeng et al. (24) found a significantly higher microbiome diversity in lung cancer than in benign nodules; however, they did not exclude patients with underlying lung diseases such as pneumonia, chronic obstructive pulmonary disease (COPD), and pulmonary fibrosis, which may have impacted the results. Therefore, we thought that the reasons for these differences might be closely related to the selection of controls and sample sources.

Microbial dysbiosis in the lung is strongly associated with the development of lung cancer (25–27). Our data indicated that *Ralstonia* significantly increased in the GGN and SN group compared to the BPD group, which suggested that *Ralstonia* may promote early lung adenocarcinoma (SN and GGN). *Ralstonia*, first discovered by Yabuuchi in Japan in 1995, is a gram-negative bacterium belonging to the *Proteobacteria* phylum (28). It was identified as the core microbiota of lung tissue (29), which is consistent with our findings. *Ralstonia* currently includes three clinically relevant species: *R. mannitolilytica*, *Ralstonia pickettii*, and *Ralstonia insidiosa* (30). *Ralstonia mannitolilytica* causes COPD exacerbation (31). *Ralstonia pickettii* was found to comprise mesothelioma-specific microbiota involved in tumor progression (32). Furthermore, Yu et al. found that lung adenocarcinoma (*n* = 6) had decreased relative abundance of *Ralstonia* than tumor tissues with squamous cell carcinoma (*n* = 25) (29). Although the results of this study differ from our findings, the main reason may be the different sample sizes as well as sample subgroups (early-stage lung cancer vs. advanced lung cancer). Next, we compared early adenocarcinoma GGN and SN and found that *Feacalibacterium*, *Serratia*, and *Blautia* were elevated in GGN but decreased in the SN group. From the perspective of clinical research, the growth rate of lung adenocarcinoma in the GGN group is comparatively slower and exhibits a more favorable prognosis when compared to the SN group (33). Therefore, we hypothesized that *Feacalibacterium* and *Blautia* may exert a protective role in the progression of GGN to SN. As reported, *Feacalibacterium* has anti-inflammatory properties and was reported to have a relatively higher abundance in lung cancer, acting in synergy with anti-PD1 in cancer treatment (34). *Blautia* was also found to have a protective effect against carcinogenic effects in intestinal cancer (35), but the clues and ideas provided by these data necessitate further experimental verification.

In addition, *Akkermansia*, *Escherichia-shigella*, and *Klebsiella* were found to be significantly enriched in the LUSC group at the genus level. In agreement with a previous study, one study also detected an increase of *Akkermansia* in lung cancer (34). However, one previous study reported a significant enrichment of *Acidovorax* in lung squamous cell carcinomas carrying TP53 mutations with a history of smoking, which is inconsistent with our results and may be related to sequencing methods, manipulation, etc. (36). In addition, *Klebsiella* also increased in LUSC. *Klebsiella pneumoniae* is the dominant strain causing lung infection in lung cancer patients and is often detected in LUSC tissue. In previous studies, *Klebsiella* was also found to be increased in squamous lung carcinoma (37), which is consistent with our findings.

Microbiota and its derived metabolite exert one of the main functions of its carcinogenesis and tumor progression (38, 39). Analysis of the function of differential metabolites can better characterize the molecular mechanisms of ESLC development. Compared with BPD, the differential metabolites of GGN were mainly enriched in phospholipase D (PLD) signaling pathway and arachidonic acid metabolism, whereas the upregulated metabolites of the SN group were mainly enriched in the steroid hormone biosynthesis pathway. PLD molecular isoforms and their hydrolysis product phosphatidic acid (PA) can activate the mTOR signaling pathway in several ways, enhancing protein synthesis in cancer cells and promoting cancer cell survival (40). In addition, arachidonic acid metabolism acts as a bridge between inflammation and cancer (41). Koundouros et al. demonstrated that arachidonic acid metabolism and oncogenic *PIK3CA* gene-associated lipid alterations are related (19). Thus, we speculate that lipid metabolism may exert an important role in GGN. In contrast, important products (e.g., estrogen) produced in the steroid hormone biosynthesis pathway which was mainly enriched in SN could stimulate epidermal growth factor receptor (EGFR) activity in lung adenocarcinoma and promote cancer cell growth; high EGFR mutation rates are associated with estrogen receptor ERβ expression (42). Therefore, the detection of ERβ expression in SN may be a predictor of the efficacy of targeted therapy in SN. Next, we found that the citrate cycle (TCA cycle) and histidine metabolism pathway were significantly up-regulated in the GGN group compared to the SN group, suggesting that the energy production of cancer cells in the GGN group was higher than that in the SN group. Enough energy contributes GGN to progress to SN.

When comparing LUSC with BPD, oxidative glutathione and reductive glutathione were heavily multiplied in LUSC. The differential metabolites were mainly enriched in the glutathione metabolism pathway. This is consistent with the study of Zhang et al., who found that increased glutathione synthesis in LUSC can generate and maintain high levels of intracellular redox homeostasis to exacerbate LUSC carcinogenesis and progression (43). Therefore, glutathione metabolism is important in squamous carcinoma and targeting the glutathione pathway may inhibit squamous carcinoma. In addition, when comparing the two groups of solid nodes (SN and LUSC), we found that the metabolic pathways in LUSC were still mainly enriched in the glutathione metabolism pathway, while the pathways upregulated in the SN group mainly included steroid hormone biosynthesis. This is consistent with the results above, and it further confirms the reliability of our results.

The results of the association analysis suggested that differential microbiota in the GGN may function through NAAG, a storage form of glutamic acid (44). The NAAG–glutamic acid cycle is a newly identified important metabolic reservoir present only in the metabolic reprogramming process of cancer cells. It was reported that glutamine production of NAAG was significantly increased in oncogenic cells compared to non-oncogenic cells. Moreover, plasma NAAG concentration was positively correlated with tumor size, and its concentration changes preceded tumor size changes, making plasma NAAG a potential biomarker for noninvasive monitoring of tumor growth (45). Therefore, our research team is collecting blood from GGN patients to analyze the plasma NAAG levels in GGN patients. The results will be presented in future research.

Next, we compared two types of adenocarcinomas (GGN and SN) and found that differential microbes enriched in GGN were negatively correlated with GNK and 3-methylhistamine. Considering that GNK and 3-methylhistamine decreased in the GGN group, we speculated that this might be related to the inertness of the GGN. Because GNK is GlcNAc kinase (46), the reduction of GNK may lead to a lower level of energy metabolism in GGN (47). 3-Methylhistamine is a degradation product of histamine. Some studies have reported that histamine and histamine receptor H1 (HRH1) induce T cell dysfunction and immunotherapy resistance, promoting tumor growth in mice and humans (48). Therefore, the reduced level of 3-methylhistamine implies that the GGN is not affected much by histamine pro-tumor growth and shows inertia.

Compared with BPD, the differential microbiota in LUSC was mainly positively correlated with creatine and N-acetylmethionine. These metabolites are all closely related to tumor promotion. In colorectal cancers, creatine synthesis enhances cancer metastasis by the upregulation of Snail and Slug expressions (49). N-Acetylmethionine is a derivative of the essential amino acid methionine (DL-methionine, Met). Methionine was utilized massively by tumor cells, which affects T-cell function. It is an immune evasion mechanism, and targeting cancer methionine signaling may provide an immunotherapy approach (50). Next, we compared LUSC with SN and confirmed again the important role of creatine in LUSC. In addition, our results showed that D-(+)-galactose was negatively correlated with differential microbiota in LUSC, which is consistent with a previous study because the research demonstrated that galactose has a protective effect on tumor growth (51).

This study utilized microbiomics and metabolomics data to construct a random forest model to identify potential biomarkers that can predict ESLC. We identified D-2-aminoadipic acid, N-[3-(aminosulfonyl) phenyl]-2,3-dihydro-1,4-benzodioxine-2-carboxamide, and 3-methylhistamine as potential markers for distinguishing GGN, SN, and LUSC from BPD, respectively. However, as all experimental samples in this study were tumor tissues, their availability is limited. In subsequent research, we plan to validate these predicted potential biomarkers using blood, sputum, or bronchoalveolar lavage fluid or to further confirm the utility of these biomarkers through studies involving cells, animals, and clinical cohorts. It was worth noticing the higher prevalence of male smokers among patients diagnosed with LUSC, potentially attributed to local lifestyle habits. Lastly, compared to the samples commonly used in previous microbiome studies on lung cancer, such as bronchial fluid, airway brushings, and sputum, our study directly utilized surgical specimens of pulmonary nodules to investigate the impact of lung microbiota in tumorigenesis. This approach effectively avoids the issue of cross-contamination between the upper and lower respiratory tract, thus providing better reliability in investigating the development mechanisms of ESLC.

## Supplementary information

In order to evaluate the complexity of the community composition and compare the differences between samples (groups), beta diversity was calculated based on weighted and unweighted unifrac distances in QIIME2.

Cluster analysis was performed with principal component analysis (PCA), which was applied to reduce the dimension of the original variables using the ade4 package and ggplot2 package in R software (Version 3.5.3).

Principal coordinate analysis (PCoA) was performed to obtain principal coordinates and visualize differences of samples in complex multi-dimensional data. A matrix of weighted or unweighted unifrac distances among samples obtained previously was transformed into a new set of orthogonal axes, where the maximum variation factor was demonstrated by the first principal coordinate and the second maximum variation factor was demonstrated by the second principal coordinate, and so on. The three-dimensional PCoA results were displayed using QIIME2 package, while the two-dimensional PCoA results were displayed using ade4 package and ggplot2 package in R software (Version 2.15.3).

To study the significance of the differences in community structure between groups, the adonis and anosim functions in the QIIME2 software were used to perform an analysis. To find out the significantly different species at each taxonomic level (phylum, class, order, family, genus, and species), the R software (Version 3.5.3) was used to perform a MetaStat and *T*-test analysis. The LEfSe software (Version 1.0) was used to perform a LEfSe analysis (LDA score threshold: 4) so as to find out the biomarkers. Furthermore, to study the functions of the communities in the samples and find out the different functions of the communities in the different groups, the PICRUSt2 software (Version 2.1.2-b) was used for function annotation analysis.

The training set and validation set used in this study were derived from a cohort of patients diagnosed with different types of pulmonary nodules, including ground glass nodules (GGN), solid nodules (SN), benign pulmonary disease (BPD), and lung squamous cell carcinoma (LUSC). The data was collected from clinical records and included various features such as metabolite levels and microbial abundance.

The entire dataset was initially split into two subsets: a training set and a validation set, following an 80/20 split. The training set, comprising 80% of the data, was used to train the Random Forest models to identify patterns associated with different pathological types. The remaining 20% of the data was set aside as the validation set to evaluate the model’s performance and ensure generalizability. Additionally, k-repeat cross-validation was used on the training set to further validate the consistency and robustness of the model during the training phase.

## Data Availability

The datasets presented in this study can be found in online repositories. The names of the repository/repositories and accession number(s) can be found below: https://www.ncbi.nlm.nih.gov/, BIOPROJECT PRJNA1012494.
