# Supplementary material for: A novel interplay between bacteria and metabolites in different early-stage lung cancer: an integrated microbiome and metabolome analysis
Source: Front Oncol. 2025 Jan 7;14:1492571. doi: 10.3389/fonc.2024.1492571 (PMC11746054; doi:10.3389/fonc.2024.1492571)
Supplement: Supplementary file 1 [file DataSheet1.docx]

**Supplementary Information**

**A novel interplay between bacteria and metabolites in different early-stage lung cancer: An integrated microbiome and metabolome analysis**

Xiaoqian Zhai^1,3^^†^, Dongqi Lin^2,3†^, Yu Fan^2,3^, Jiabi Zhang^4^, Yiyun Lin^5^, Yuqing Wang^6^, Qinghua Zhou^2,3^, Xi Zheng^2,3^^*^

^*^Corresponding author: rmhahea@ucl.ac.uk

^†^Xiaoqian Zhai, Dongqi Lin are regarded as co-first authors.

**This PDF file includes:**

Figures. S1 to S2

**Fig S1**

**
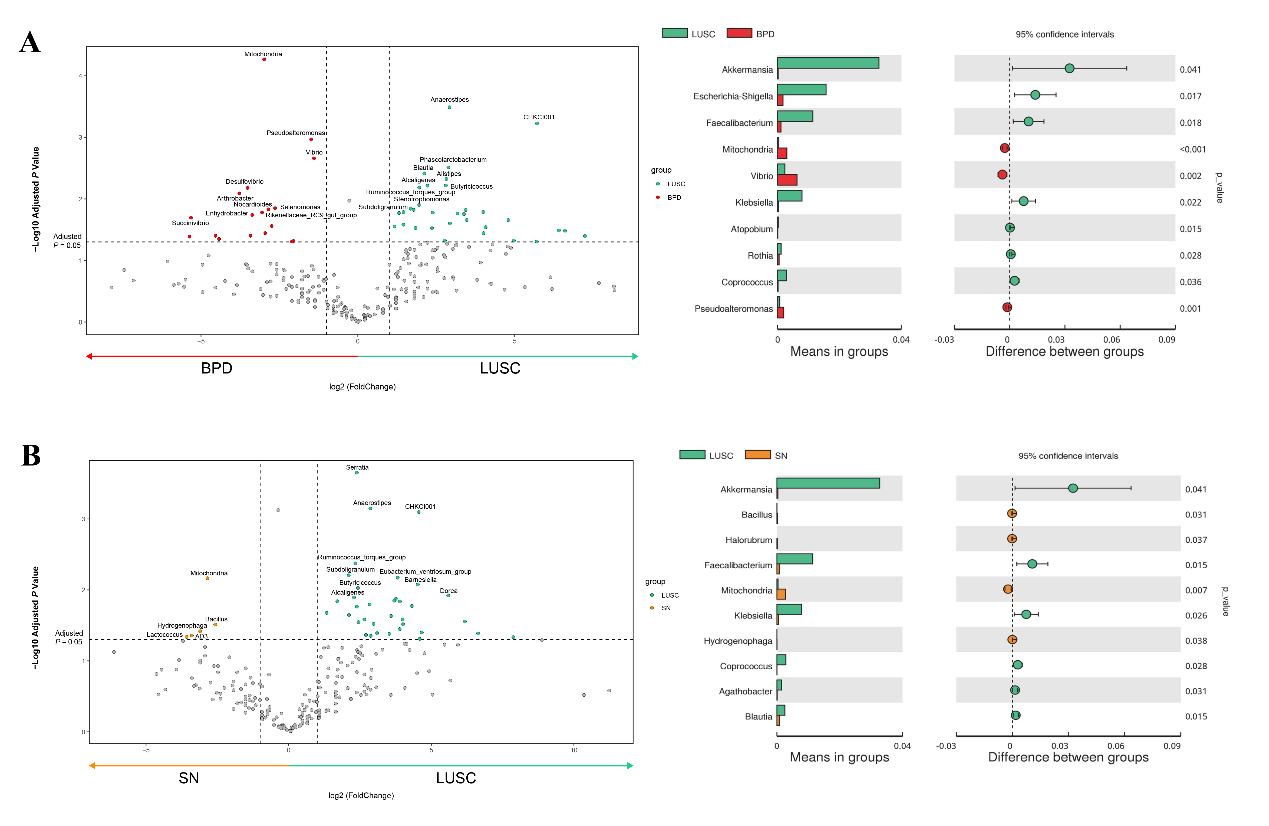
**

When compared with BPD groups, *Akkermansia*, *Escherichia-shigella* and *Klebsiella* were found to be significantly enriched in the LUSC group at the genus level, while *Vibrio, Pseudoalteromonas*, and *Atopobium* were significantly enriched in the BPD group (Fig.S1A). Next, we compared LUSC with lung adenocarcinoma (SN) and found that *Bacillus, and Hydrogenophaga* were significantly enriched in the SN group, whereas *Akkermansia, Feacalibacterium* and *Klebsiella* were significantly enriched in the LUSC group (Fig.S1B).

**Fig S2**

**
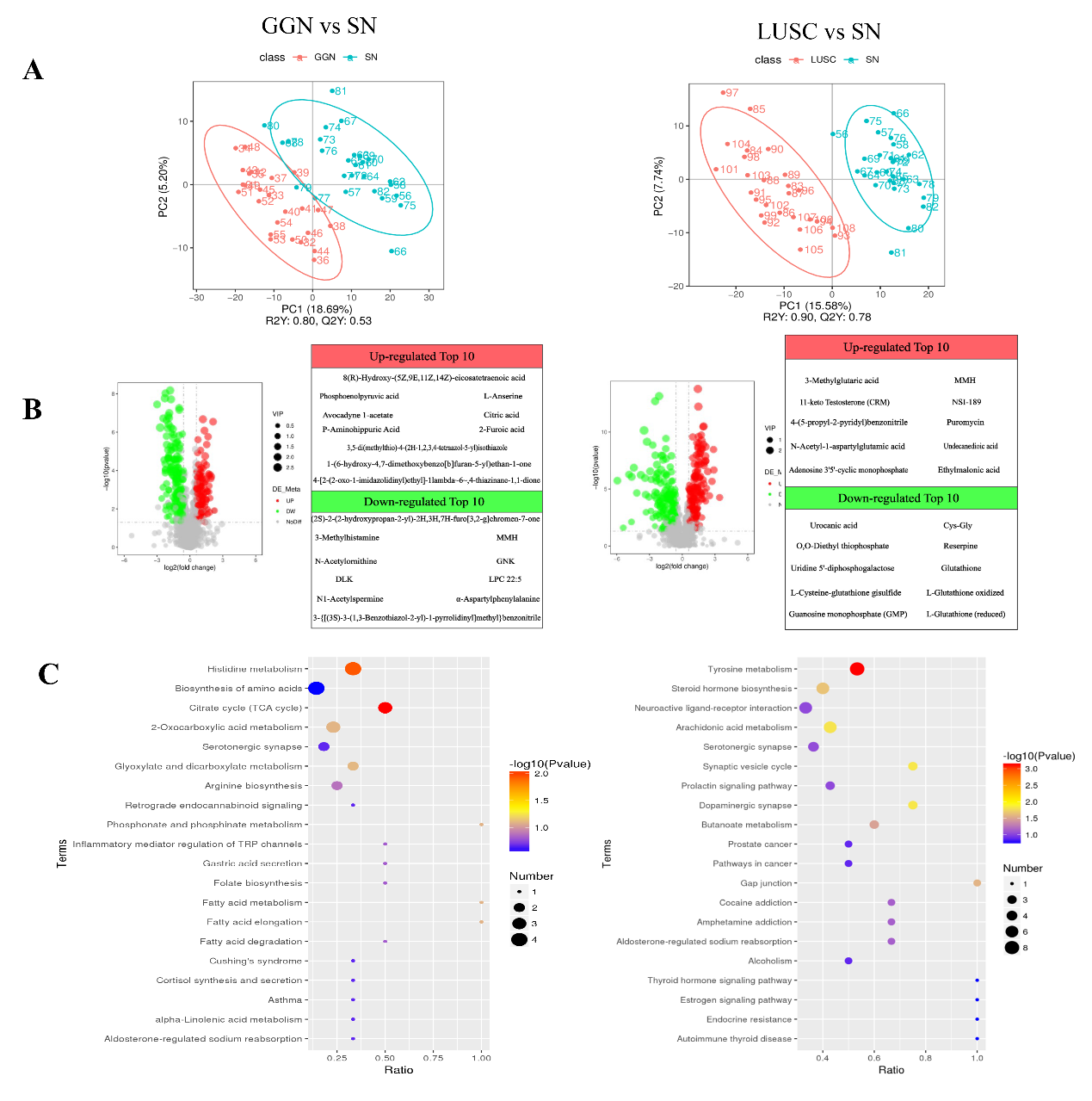
**

We validated the metabolomic data using the OPLS-DA model and found that the metabolic data were comparable between the lung nodule pairs (Fig. S2A). We compared the two groups of early adenocarcinoma (GGN and SN) and found that 122 differential metabolites were up-regulated and 189 metabolites were down-regulated in the GGN group. Among them, the top 3 differential metabolites of the GGN group were 8(R)-Hydroxy-(5Z,9E,11Z,14Z)-eicosatetraenoic acid (fold change=4), L-Anserine (fold change=4) and Phosphoenolpyruvic acid (fold change= 4) (Fig. S2B). KEGG enrichment analysis revealed that the pathways of up-regulated metabolite enrichment in the GGN group were the Citrate cycle (TCA cycle), Histidine metabolism (Fig. S2C). Next, we compared SN (adenocarcinoma) and LUSC (solid nodules on CT) and found 196 metabolites up-regulated, while 161 down-regulated in the SN group. The top 3 differential metabolites were Puromycin (fold change=8), Adenosine 3'5'-cyclic monophosphate (fold change=8), and Ethylmalonic acid (fold change=8) (Fig. S2B). The KEGG pathways enriched in the SN group included Tyrosine metabolism, Arachidonic acid metabolism, and Steroid hormone biosynthesis (Fig. S2C). Glutathione metabolism, Pyrimidine metabolism, and Fatty acid biosynthesis were enriched in LUSC.

**Supplementary Figure legends**

Fig S1. Comparison of differential microflora between LUSC and BPD or SN, respectively. At the genus level, volcano plot using T-test analysis showing the differential flora (fold change> 2) of BPD and LUSC (A); SN and LUSC (B); And bar graph showing the mean abundance and *P* value of BPD and LUSC (A); SN and LUSC (B).

Fig S2. Differential metabolites and pathways of GGN and LUSC when compared with SN, respectively. A. PLS-DA score scatterplot of GGN and LUSC when compared with SN, respectively; B. Volcano plot showing the differential metabolites of GGN and LUSC when compared with SN, respectively. Set thresholds: VIP > 1.0, FC > 1.5 or FC < 0.667 and P value< 0.05). C. Bubble plots of KEGG enrichment of GGN and LUSC when compared with SN, respectively.
